# Correlated Increase of High Ocean Waves and Winds in the Ice-Free Waters of the Arctic Ocean

**DOI:** 10.1038/s41598-018-22500-9

**Published:** 2018-03-14

**Authors:** Takuji Waseda, Adrean Webb, Kazutoshi Sato, Jun Inoue, Alison Kohout, Bill Penrose, Scott Penrose

**Affiliations:** 1 The University of Tokyo, Graduate School of Frontier Sciences, Kashiwa, 277-8563 Japan; 2 National Institute of Polar Research, Arctic Environment Research Center, Tachikawa, 190-8518 Japan; 3 National Institute of Water and Atmospheric Research, Hydrodynamics, Christchurch, 8011 New Zealand; 4 P.A.S. Consultants Pty Ltd., Panton Hill, 3759 Australia; 5 Present Address: Kyoto University, Disaster Prevention Research Institute, Kyoto, 611-0011 Japan; 6 Present Address: Kitami Institute of Technology, Kitami, 090-8507 Japan

## Abstract

The long-term trend of extreme ocean waves in the emerging ice-free waters of the summer Arctic is studied using ERA-Interim wave reanalysis, with validation by two drifting wave buoys deployed in summer 2016. The 38-year-long reanalysis dataset reveals an increase in the expected largest significant wave height from 2.3 m to 3.1 m in the ice-free water from the Laptev to the Beaufort Seas during October. The trend is highly correlated with the expected increase in highest wind speed from 12.0 m/s to 14.2 m/s over the ice-free ocean, and less so with the extent of the ice-free water. Since the storms in this area did not strengthen throughout the analysis period, the increase in the expected largest significant wave height follows from the enhanced probability of storms in ice-free waters, which is pertinent to the estimation of extreme sea conditions along the Northern Sea Route.

## Introduction

The area of ice-covered sea in the Arctic Ocean during the boreal summer has reduced by a few million square kilometres in previous decades^1^, resulting in the emergence of ice-free waters on which the wind can generate waves. In latitudes north of 70°N, the mean significant wave height during summer exceeds 3 m in some areas^2^. As the entire Arctic Ocean is expected to be ice-free by 2050, the wave height is projected to correspondingly increase^3^. In the last decades, the waves in the Arctic Ocean have gradually increased as substantiated by numerous studies based on satellite observations and reanalysis^4–6^. Collins *et al*.^7^ reported a significant wave height of almost 5 m in the Barents Sea based on the analysis of the ship motion. During the R/V Sikuliaq cruise in 2015, a wave buoy measured waves reaching 5 m in the Beaufort sea^8^. Over 200 vessels have used the Northern Sea Route (NSR) since 2011. While these vessels must comply with the ice-class rules, they also need to take into consideration the increasing height of the ocean waves in the Arctic Ocean. In the presence of high waves and low temperatures, sea-spray icing may also become a serious problem.

Given the expected increase of ship traffic in the Arctic Ocean, estimation of the largest waves in ice-free water is a crucial issue yet to be addressed. Assuming the probability density function (p.d.f.) of the significant wave height *H*_*s*_ in the ice-free waters is approximated by a Weibull distribution, $P({H}_{s})=\exp \{-{(\frac{{H}_{s}}{{c}_{{H}_{s}}})}^{k}\}$, where *k* is a shape parameter, then the maximum significant wave height

1 $${H}_{s}^{max}={c}_{{H}_{s}}{(\mathrm{ln}N)}^{\frac{1}{k}},$$

which is valid for a large value of *N*, where *N* is the number of data samples^9^. This simple relationship indicates that the largest *H*_*s*_ in ice-free waters is related to the distribution of *H*_*s*_ represented by the scale parameter ${c}_{{H}_{s}}$ and the extent of the ice-free waters, which is represented in terms of *N*. Thomson *et al*.^10^ estimated a 0.01 m/year increase of the scale parameter ${c}_{{H}_{s}}$ in the Beaufort and Chukchi seas based on 23-year reanalysis data of the wave field, and attributed this to the enlarged area of ice-free waters, which extends the fetch, regardless of the variation in the wind field^11^. In contrast, the maximum *H*_*s*_ increases with *N* as well according to equation (1), with the physical implication being that as the area of the ice-free water increases, the chances of encountering a large wave also increase.

For a given value of *N*, the observed maximum ${H}_{s}^{max}$ is itself a stochastic variable, whose expectation or mean can be expressed as

2 $$E[{H}_{s}^{max}]={\int }_{0}^{\infty }[1-P{({H}_{s})}^{N}]d{H}_{s}.$$

From observational or reanalysis data, however, it is more practical to invoke an ergodic hypothesis and estimate the mean from the time average

3 $$E[{H}_{s}^{max}]\approx \frac{1}{M}\sum _{i=1}^{M}{H}_{s}^{max}({t}_{i}),$$

where *M* is the number of available data samples for a given time interval. While a point-by-point statistics in the ice-covered ocean requires a careful analysis^12^, the proposed method allows a straightforward implementation, and is also directly related to the encounter wave statistics for the NSR vessels.

For observing the long-term trend of the largest significant wave height in the Arctic Ocean, the expectation of the area-maximum *H*_*s*_ is estimated using equation (3). Considering that the sea-ice extent starts to decrease in August and recovers in October, the monthly means are calculated for August, September and October corresponding to the melting, ice-free and freezing periods, respectively. The ERA-interim reanalysis data^13^ is used for the analysis in the Laptev, East Siberian, Chukchi and Beaufort Seas. The underlying assumption of the analysis is to disregard the changes of the distribution function *P*(*H*_*s*_) during a particular month of the analysis period, with a similar analysis conducted for the area of ice-free water, and the wind speed at a height of 10 m. A correlation analysis of the wave height, the area of ice-free water, and the wind speed is also conducted.

## Results

### Validation of the reanalysis wave field

The ERA-Interim reanalysis is a global atmospheric, wave and ice data assimilation system beginning in 1979 and continuing in near real-time^13^. The spatial resolution of the wave model in the Arctic Ocean is around 110 km in longitude and 110 km in latitude, with wave spectra available for 24 directions and 30 frequencies. The relatively coarse grid resolution prevents the resolution of the fine-scale structure of the ice edge in the Arctic Ocean. While the recent reanalysis assimilates significant-wave-height data from an altimeter on the Jason-2 satellite, its orbital inclination of 66.038° precludes measurement of Arctic Ocean waves, implying the Artic wave field of the ERA-Interim reanalysis system is essentially lacking any data assimilation in recent years. Since ERS-1/2 were assimilated from Aug. 1991 to Jul. 2003, and ENVISAT from Jul. 2003 to Aug. 2012, 1991 and 2011 marks the transition between assimilation free and assimilated periods, which could be critical in the trend analysis. The sea-surface temperature (SST) and the ice concentration data assimilation systems have utilized the Operational Sea Surface Temperature and Sea-Ice Analysis (OSTIA) data in recent years. As Dee *et al*.^13^ report that the surface wind speed depends strongly on the SST and extent of the ice coverage in the polar region, uncertainties in the OSTIA sea-ice cover and SST affect the magnitude of the surface wind speed. Therefore, without data assimilation, the accuracy of the ERA-interim wave field remains uncertain.

**Figure 1:**
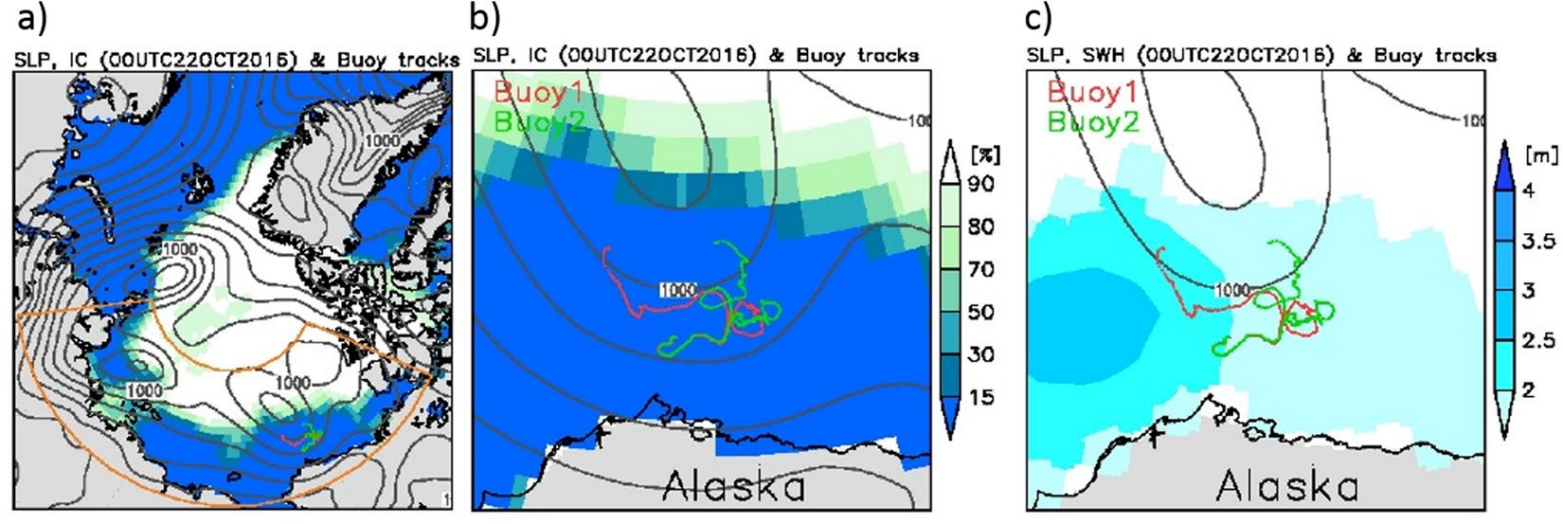
(**a**) Buoy trajectories, sea-ice concentration (colour shading) and sea-level pressure (SLP) (contours) on 22 October 2016. The region bounded by the orange line indicates the area analyzed here. (**b**) An enlarged image of the buoy trajectories with the sea-ice concentration (colour shading) and SLP (contours) on 22 October 2016. (**c**) An enlarged image of the buoy trajectories with the significant wave height (colour shading) and SLP (contours) on 22 October 2016. The SLP, *H*_*s*_ and sea-ice concentration are from ERA-Interim reanalysis data. The Grid Analysis and Display System (GrADS) version 2.0.2 (http://cola.gmu.edu/grads/) was used to create the maps in this figure.

The ERA-Interim wave field in the Chukchi and Beaufort Seas, interpolated on the wind and wave collocated 0.75 degrees regular grid, was validated by a 2016 wave observation campaign by The University of Tokyo. During the Mirai cruise (MR16-06) of the Japan Agency for Marine-Earth Science and Technology from 22 August to 5 October 2016, two drifting-type wave buoys (WII: Waves In Ice buoys) were deployed off Point Barrow on 10 September. The two buoys remained within an area of a few hundred square kilometres, and successfully collected wave data before contact was lost with both on 2 November 2016. The tracks of the two buoys, which are indicated by red and green trajectories in Fig. 1, remained in close proximity to one another until 19 September when a storm separated the buoys by approximately 50 km, before further separating during the 18 October storm by 250 km to 300 km.

**Figure 2:**
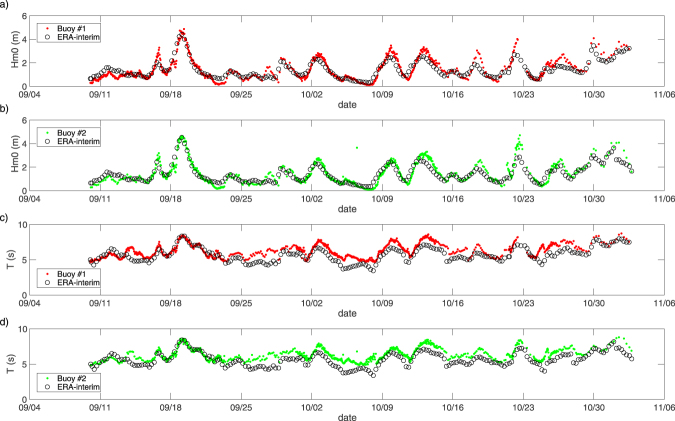
The ERA-interim wave field (○) together with (**a**) the significant wave height of buoy 1 (red ●), (**b**) the significant wave height of buoy 2 (green ●), (**c**) the mean wave period of buoy 1 (red ●), and (**d**) the mean wave period of buoy 2 (green ●).

The ERA-Interim *H*_*s*_ at the buoy locations compares well with the observations (Fig. 2). Since the spatial resolution is coarse and time intervals are sparse, the nearest-neighbour interpolation was applied to the ERA-Interim data. The correlation coefficient between the ERA-Interim data and observed *H*_*s*_ is 0.91 for both buoys, but 0.78 and 0.76 between the mean wave periods of the ERA-Interim data and the observations for buoys 1 and 2, respectively. While the timings of the storm events are well reproduced, the magnitudes of *H*_*s*_ are slightly under-predicted during October. The reproducibility of mean wave period is not as good as that of significant wave height, and the differences between ERA-Interim mean wave period and observed energy period, *T*_0,−1_ tend to be larger for smaller wave heights. The error metrics are summarized in Table S1. A similar comparison was made with measurements obtained with the Surface Wave Instrument Float with Tracking (SWIFT) buoy in 2014^11^, which was deployed by the University of Washington and measured wave heights from 27 July to 28 September 2014 in the Chukchi and Beaufort Seas, to find a correlation coefficient with ERA-Interim data of 0.91, which is the same as for our 2016 observations.

**Figure 3:**
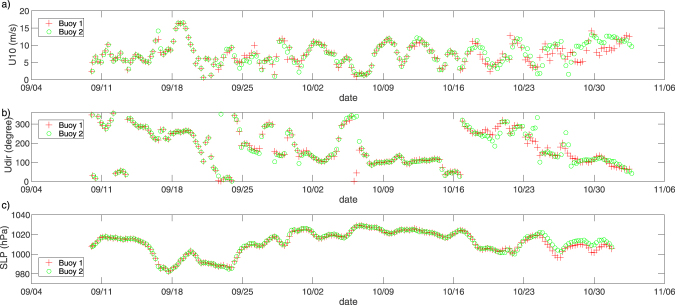
ERA-Interim data at the locations of buoys 1 (red) and 2 (green) for (**a**) 10-m wind speed, (**b**) 10-m wind direction, and (**c**) sea-level pressure.

Large wave events were observed, for example, on 19 September, when buoys 1 and 2 recorded a *H*_*s*_ of 4.86 m and 4.63 m, respectively, which is comparable to the highest wave directly observed in this region by Thomson *et al*.^8^. During this time, an Arctic cyclone developed off Point Barrow (Fig. S1)^14^ to the north of the buoy locations. As the location of the large wave height was recorded south of the cyclone centre, which is relatively widespread, the largest significant wave height in this region may even have been larger than the buoy observation. The *H*_*s*_ of the ERA-Interim data and the buoys agree quite well during this storm. However, during the October 22 storm (Fig. 1), when buoy 2 recorded a significant wave height of 4.7 m, the reanalysis fails to reproduce the observed wave height. For both the 19 September and 22 October storms, the pressure minimums were located to the north of the buoys, and, therefore, the wind direction was predominantly from the west (Fig. 3). The wind speeds were relatively high during both storms at around 16 m/s and 13 m/s, respectively. Compared with the 19 September condition, the area of the ice-free waters on 22 October has substantially reduced. However, the cause of the difference of the observed and reanalysis *H*_*s*_ for the October event is not because of the coarse resolution of ERA-Interim. The reproducibility of the October event did not improve with a 16 km high-resolution modeling that was conducted in a related study, and the reason for the discrepancy is likely the lack of accuracy of the wind. Distinct from the storm event observed during the Sikuliaq cruise in 2015 which was associated with a high-pressure system and an Easterly wind, both the September and the October events in 2016 were associated with an Arctic Cyclone. The wind was blowing from the west, and the sea ice extended south into the west of the Chukchi sea. However, the effective fetch was not limited by the sea ice in the upwind direction as the scale of the storm was comparable to the open water in the Chukchi and the Beaufort Seas (Fig. 1).

### The trend of the expected largest significant wave height in the ice-free waters

The wave, wind and ice fields are analyzed within a domain including the Laptev, East Siberian, Chukchi and Beaufort Seas (96.75 E to 113.25 W and 68.25 N to 81.75 N), which is the area bounded by the orange line in Fig. 1. The area of ice-free water in the domain varies during the summer season and enlarges over the analyzed 38 years. Note that ERA-Interim treats any area with a sea ice cover over 30% as without waves and the rest with ice cover less than 30% as ice-free. In the rest of this paper, we will consider grid-points where the wave parameter is undefined as areas covered with ice. The domain was chosen such that the boundary is either land or ice, even during the largest retreat of sea ice in 2012, except for the Bering Strait. The percentage of the ice-free water area to the total area of the domain including land and ice, *ρ*, is equivalent to the ratio of the number of the ERA-Interim grid points in the completely ice-free water and the total number of grid points in the domain area; ${\rho }\approx \frac{{N}_{icefree}}{{N}_{total}}$. Thus, this index *ρ* is related to the number of ice-free data points *N* in the domain (see equation 1). The 6-h significant wave height and wind speed at 10 m in August, September, and October are analyzed.

The August, September, and October monthly areal averages of *H*_*s*_ and *U*_10_ are calculated, along with the 38-year trends. The August, September and October climatological values of *H*_*s*_ are 0.85 ± 0.11 m, 1.07 ± 0.12 m and 1.26 ± 0.17 m, respectively. The highest mean wave height and greatest variability occur in October. In Table 1, the linear trend of the mean *H*_*s*_ and the scaling parameter of the Weibull fit are shown. On average, the linear trend of *H*_*s*_ is around 5 mm/year, and that of the scaling parameter of the Weibull distribution is of the same magnitude. The linear trend of the scaling parameter ${c}_{{H}_{s}}$ is about half that estimated by Thomson *et al*.^10^ which is based on 23-year reanalysis data. The difference of the data length may account for this difference implying the acceleration of the increasing trend of the average wave height in the Arctic Ocean. We can state that since 1979, the average *H*_*s*_ has increased by about 20%, corresponding to 0.15 m in August, 0.19 m in September, and 0.20 m in October. The estimate falls within the range of the increasing trend, 0.3–0.8%/year, detected by Wang *et al*.^6^. The wind speed over the ice-free waters, however, does not show any noticeable trend (Table 1). The August and September *U*_10_ decreases by about 0.06 m/s and 0.12 m/s, respectively, and increases by about 0.17 m/s in October over the 38 years investigated, which amount to only a few percent of the climatological wind speeds of 6.0 m/s in August, 6.6 m/s in September, and 7.1 m/s in October. An independent study by Wang *et al*.^6^ also detects no statistically significant trend of the areal mean wind speed.

**Table 1:** Linear trends of the mean values and the scaling parameter ${c}_{{H}_{s}}$ of the Weibull fit to *H*_*s*_, and the linear trend of the mean values of the wind speed *U*_10_.

Trends	mean *H*_*s*_ (m/year)	${c}_{{H}_{s}}$ (m/year)	mean *U*_10_ (m/s/year)
August	0.004 (R2 = 0.17)	0.0039 (R2 = 0.14)	−0.0015 (R2 = 0.0017)
September	0.0051 (R2 = 0.23)	0.0060 (R2 = 0.24)	−0.0031 (R2 = −0.003)
October	0.0054 (R2 = 0.12)	0.0049 (R2 = 0.049)	0.0045 (R2 = 0.013)

**Figure 4:**
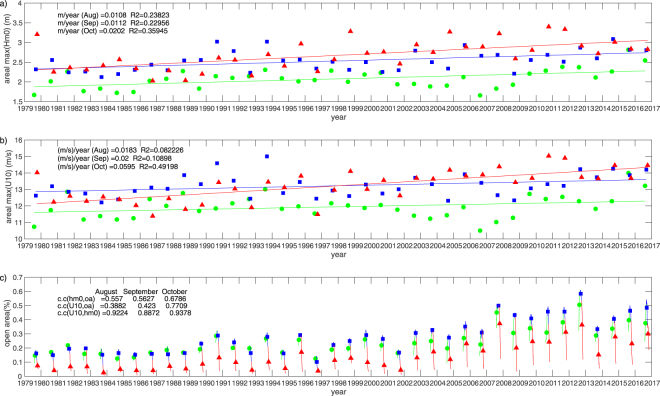
(**a**) The annual $E[{H}_{s}^{max}]$ are plotted for August (), September () and October () from 1979 to 2016. The linear trends are indicated by green (August), blue (September) and red lines (October); (**b**) The annual *E*[*U*_10_] and trend lines, with nomenclature the same as (**a**); (**c**) The annual *E*[*ρ*]. The solid lines indicate the change of *ρ* from August to October.

On the contrary, the maximum *H*_*s*_ in the ice-free water has clearly an increasing trend, where Fig. 4a shows the expectation of the maximum *H*_*s*_ in the ice-free water according to equation (3). The inter-annual variation is large as inferred from the scatter of the annual $E[{H}_{s}^{max}]$ (green circles, blue squares, and red triangles) along the linear trends (green, blue and red lines) for August, September and October, respectively. The increasing trend is largest in October (Table 2), with an increase of almost 30% since 1979, which is more than a 70 cm increase from *H*_*s*_ ≈ 2 m. For the other months, the increase is around 20% or 40 cm from 1979 to 2016. The robustness of the trend was tested using the homogenization tool, and was confirmed that the mixture of the assimilated and assimilation-free periods did not affect the detected trend estimate, see Fig. S2^15,16^. As the ice starts to melt in July and August, the area of the ice-free water *E*[*ρ*] was little more than 10% in the 1980s (Fig. 4c); September had a similar percentage of ice-free water. However, in October, the area reduced to less than 10%. The area of ice-free water gradually started to increase in the 1990s and 2000s, when the percentage of ice-free water in September increased to around 30%, and then rapidly increased in the 2010s, reaching around 60% in 2012, which is the largest ice retreat to date. The correlation coefficient between the area of the ice-free water *E*[*ρ*] and the annual $E[{H}_{s}^{max}]$ is 0.56 for August and September, and 0.68 for October. Therefore, to a certain extent, the long-term trend of the increase of the maximum significant wave height can be explained by the increase in the area of the ice-free water.

**Table 2:** Linear trends of the maximum values of *H*_*s*_ and *U*_10_.

Trends	max of *H*_*s*_ (m/year)	increase over 38 years	max of *U*_10_ (m/s/year)	increase over 38 years
August	0.0108 (R2 = 0.24)	0.41 m	0.0183 (R2 = 0.082)	0.70 m/s
September	0.0112 (R2 = 0.23)	0.43 m	0.0200 (R2 = 0.11)	0.76 m/s
October	0.0202 (R2 = 0.36)	0.77 m	0.0595 (R2 = 0.49)	2.26 m/s

A possible reason for the enhancement in wave height as the area of open water expands is the increase in the effective fetch *F*^11^ on which the significant wave height depends following

4 $$(\frac{g{H}_{s}}{{U}_{10}^{2}})\propto {(\frac{gF}{{U}_{10}^{2}})}^{\alpha },$$

where *α* is typically around 1/2. Hence, the growth of the significant wave height depends on both the fetch *F* and the wind speed. While the retreat of sea ice during summer may enhance the strength of Arctic cyclones as a result of the thermal contrast between the ocean and atmosphere^17^, the extent is not yet evident to which the wind speeds over the ice-free water are enhanced. The ERA-Interim wind speed at 10-m altitude over the ice-free water is shown in Fig. 4b. The grid points of winds over ice are detected from the undefined values of the collocated wave height, and are excluded. The annual $E[{U}_{10}^{max}]$ varies similarly to the annual $E[{H}_{s}^{max}]$, where the greatest positive trend is found for October (Table 2). For August and September, the $E[{U}_{10}^{max}]$ increased by around 70 cm/s from 1979 to 2016, while the October $E[{U}_{10}^{max}]$ increased by around 2.3 m/s, which accounts for the increase of the expected maximum wind speed in the ice-free waters from 12 m/s to 14.2 m/s in October. Moreover, the inter-annual variation seems to follow a similar change between $E[{H}_{s}^{max}]$ and $E[{U}_{10}^{max}]$. Indeed, extremely high correlations of 0.92 for August, 0.89 for September and 0.94 for October are detected. In contrast, the correlation between *E*[*ρ*] and $E[{U}_{10}^{max}]$ is 0.39 for August, 0.42 for September, and 0.77 for October, and hence are relatively lower. It is, therefore, puzzling that while the wind speed seems to affect the significant wave height directly in the ice-free waters, it itself is not affected as much as one would have conjectured by the enlarged area of the ice-free waters.

### Possible causes of the increase in the maximum wave height: how does the wind speed intensify?

To further clarify the cause of the positive trend of the maximum significant wave height in the Arctic Ocean, a partial correlation analysis was conducted. As depicted in Fig. 4, the inter-annual variation is the dominant signal of the $E[{H}_{s}^{max}]$ and $E[{U}_{10}^{max}]$. A 7-year moving average is used as a low-pass filter to separate the low from the high frequency components,

5 $$E[{H}_{s}^{max}]=\,\langle E[{H}_{s}^{max}]\rangle +\,(E[{H}_{s}^{max}])^{\prime} $$

6 $$E[{U}_{10}^{max}]=\,\langle E[{U}_{10}^{max}]\rangle +(E[{U}_{10}^{max}])^{\prime} $$

7 $$E[\rho ]=\langle E[\rho ]\rangle +(E[\rho ])^{\prime} ,$$

where the low-pass filter is denoted by <> and the high-pass filter by ()′. The partial correlations are applied to the low-pass filtered $\langle E[{H}_{s}^{max}]\rangle$, $\langle E[{U}_{10}^{max}]\rangle$ and 〈*E*[*ρ*]〉, and high-pass filtered $(E[{H}_{s}^{max}])^{\prime}$, $(E[{U}_{10}^{max}])^{\prime}$ and (*E*[*ρ*])′ (Table 3).

**Table 3:** Partial correlations among high-pass-filtered expected-area maxima of wave height $(E[{H}_{s}^{max}])^{\prime}$, wind speed $(E[{U}_{10}^{max}])^{\prime}$, and the ice-free water area (*E*[*ρ*])′ (left column), and low-pass-filtered expected-area maxima of wave height $\langle E[{H}_{s}^{max}]\rangle$, wind speed $\langle E[{U}_{10}^{max}]\rangle$, and ice-free water area 〈*E*[*ρ*]〉 (right column).

August (high)	${\boldsymbol{(}}{\boldsymbol{E}}{\boldsymbol{[}}{{\boldsymbol{H}}}_{{\boldsymbol{s}}}^{{\boldsymbol{m}}{\boldsymbol{a}}{\boldsymbol{x}}}{\boldsymbol{]}}{\boldsymbol{)}}{\boldsymbol{^{\prime} }}$	${\boldsymbol{(}}{\boldsymbol{E}}{\boldsymbol{[}}{{\boldsymbol{U}}}_{{\bf{10}}}^{{\boldsymbol{m}}{\boldsymbol{a}}{\boldsymbol{x}}}{\boldsymbol{]}}{\boldsymbol{)}}{\boldsymbol{^{\prime} }}$	August (low)	${\boldsymbol{\langle }}{\boldsymbol{E}}{\boldsymbol{[}}{{\boldsymbol{H}}}_{{\boldsymbol{s}}}^{{\boldsymbol{m}}{\boldsymbol{a}}{\boldsymbol{x}}}{\boldsymbol{]}}{\boldsymbol{\rangle }}$	${\boldsymbol{\langle }}{\boldsymbol{E}}{\boldsymbol{[}}{{\boldsymbol{U}}}_{{\bf{10}}}^{{\boldsymbol{m}}{\boldsymbol{a}}{\boldsymbol{x}}}{\boldsymbol{]}}{\boldsymbol{\rangle }}$
(*E*[*ρ*])′	0.077	−0.474	〈*E*[*ρ*]〉	0.781	−0.687
$(E[{H}_{s}^{max}])^{\prime}$	—	0.776	$\langle E[{H}_{s}^{max}]\rangle$	—	0.947
**September (high)**	$({\boldsymbol{E}}[{{\boldsymbol{H}}}_{{\boldsymbol{s}}}^{{\boldsymbol{\max }}}])^{\prime}$	$({\boldsymbol{E}}[{{\boldsymbol{U}}}_{{\bf{10}}}^{{\boldsymbol{\max }}}])^{\prime}$	**September (low)**	$\langle {\boldsymbol{E}}[{{\boldsymbol{H}}}_{{\boldsymbol{s}}}^{{\boldsymbol{\max }}}]\rangle$	$\langle {\boldsymbol{E}}[{{\boldsymbol{H}}}_{{\boldsymbol{s}}}^{{\boldsymbol{\max }}}]\rangle$
(*E*[*ρ*])′	−0.014	0.665	〈*E*[*ρ*]〉	0.755	−0.554
$(E[{H}_{s}^{max}])^{\prime}$	—	0.679	$\langle E[{H}_{s}^{max}]\rangle$	—	0.921
**October (high)**	$({\boldsymbol{E}}[{{\boldsymbol{H}}}_{{\boldsymbol{s}}}^{{\boldsymbol{\max }}}])^{\prime}$	$({\boldsymbol{E}}[{{\boldsymbol{U}}}_{{\bf{10}}}^{{\boldsymbol{\max }}}])^{\prime}$	**October (low)**	$\langle {\boldsymbol{E}}[{{\boldsymbol{H}}}_{{\boldsymbol{s}}}^{{\boldsymbol{\max }}}]\rangle$	$\langle {\boldsymbol{E}}[{{\boldsymbol{U}}}_{{\bf{10}}}^{{\boldsymbol{\max }}}]\rangle$
(*E*[*ρ*])′	−0.043	0.380	〈*E*[*ρ*]〉	−0.65	0.845
$(E[{H}_{s}^{max}])^{\prime}$	—	0.868	$\langle E[{H}_{s}^{max}]\rangle$	—	0.947

High partial correlations are found between the low-pass filtered wave height and wind speed, $\langle E[{H}_{s}^{max}]\rangle$ and $\langle E[{U}_{10}^{max}]\rangle$, excluding the influence of 〈*E*[*ρ*]〉, and amount to 0.95 for August, 0.92 for September and 0.95 for October. Conversely, the partial correlations between ice-free water area 〈*E*[*ρ*]〉 and $\langle E[{H}_{s}^{max}]\rangle$ or $\langle E[{U}_{10}^{max}]\rangle$ are much smaller, and may even take a negative value. Therefore, the long-term positive trend of the maximum wave height $\langle E[{H}_{s}^{max}]\rangle$ is closely related to the long-term positive trend of the maximum wind speed over the ice-free waters $\langle E[{U}_{10}^{max}]\rangle$ angle, but less so to the ice-free water area 〈*E*[*ρ*]〉.

While the correlation between the high-pass filtered wave height $(E[{H}_{s}^{max}])^{\prime}$ and wind speed $(E[{U}_{10}^{max}])^{\prime}$ (0.78 for August, 0.68 for September, 0.87 for October) is not as high as that of the low-pass filtered data, it is much more pronounced than the correlation between the high-pass filtered ice-free area (*E*[*ρ*])′ and the wave height $(E[{H}_{s}^{max}])^{\prime}$ (<0.1). Therefore, while the inter-annual variation of the significant wave height is not directly correlated with the inter-annual variation of the ice coverage in the Arctic Ocean, $(E[{U}_{10}^{max}])^{\prime}$ is correlated well with (*E*[*ρ*])′ in September, but relatively weakly in other months. In summary, the correlation of interannual variation of the maximum wind speed $(E[{U}_{10}^{max}])^{\prime}$ and the maximum wave height $(E[{H}_{s}^{max}])^{\prime}$ is reasonably large, but other correlations do not show a systematic tendency.

Overall, the partial correlation analysis reveals that the area-maximum significant wave height in the Arctic ice-free waters is not directly influenced by the ice coverage for both long-term and inter-annual variations, but is strongly affected by the maximum wind speed in the ice-free water area. Whether this is related to the change in storm intensity in the past several decades is discussed below.

## Discussion

Analysis of the ERA-Interim wave field in the Arctic ice-free waters from 1979 to 2016 indicates a positive trend of the expected area-maximum wave height $E[{H}_{s}^{max}]$, which amounts to 77 cm in October. It is also shown that $E[{H}_{s}^{max}]$ is strongly correlated with the expected area-maximum wind speed $E[{U}_{10}^{max}]$, but less so with the ice-free water area *E*[*ρ*]. That $E[{H}_{s}^{max}]$ increases in time may follow from (1) the increase in the ice-free water fetch, (2) the increase in the storm intensity, or (3) the increase in the encounter probability with high wind speeds. These points are elaborated below.

For flow from the land or ice to ice-free water, waves are generated and gradually grow with distance according to the so-called fetch laws derived from observations^18,19^, which serve as benchmarks in the tuning of third-generation wave models. However, it is rare to find meteorological conditions with a well-defined fetch in the wind direction, including near the coast, as the fetch geometry can be rather complicated in an enclosed basin. Recent findings of wave propagation at the marginal ice-zone detected by SAR observations imply that the determination of a well defined fetch in the partially ice-covered sea can be laborious^20–22^. For the case of waves under a hurricane, an “equivalent fetch” replaces the physical distance over which the flow traverses (see the extensive review by Young^23^). As the ice starts to melt and ice-free water emerges in the Arctic Ocean^24^, the wave behaviour at this early stage may resemble that in an enclosed basin. Eventually, the area of ice-free water surpasses the scale of the storm, with conditions resembling that of waves under a hurricane. With the increase in the area of ice-free water in time, the lake-like conditions transition into hurricane-like conditions in the open waters, so that the ice-free water distance may no longer be the most relevant parameter. It is, therefore, unsurprising that $E[{H}_{s}^{max}]$ does not correlate well with the area of ice-free water. Thomson and Rogers^11^ show that the wind waves generated by the local wind approximately follow the fetch law. However, if we limit our attention to the largest waves in the ice-free water, the ice-free water distance as represented by the area of ice-free water does not correlate well with the wave height. The fetch effect, therefore, is not the main cause of the positive trend of $E[{H}_{s}^{max}]$ in the ice-free water.

**Figure 5:**
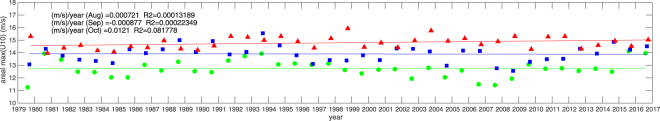
The annual *E*[*U*_10_] analyzed over areas including land and ice-covered seas, with trends plotted for August (), September () and October () from 1979 to 2016. The linear trends are indicated by green (August), blue (September) and red lines (October).

Another possibility is a long-term positive trend of the wind speed. However, Koyama *et al*.^25^ investigated the NCEP/NCAR reanalysis dataset from 1979 to 2014 and show that there is no coherent trend in storm frequency or intensity despite the increasing loss of sea ice during summer. This is consistent with Sato and Inoue^26^, who show that the trend in sea-level pressure is almost negligible in contrast to the strong decrease in ice concentration. In the literature, there is also no evidence of the increase of the intensity or frequency of storms in the Arctic Ocean. This is confirmed by analyzing the ERA-Interim wind speed in the Laptev, East Siberian, Chukchi and Beaufort Seas, including the land and ice-covered areas, which shows little positive trend in the maximum wind speed (Fig. 5), and is at most 1.2 cm/s in October, and less than 1 mm/s for the other months. As the R-squared values of the linear fit are extremely small, there is no indication of the strengthening or enhancement of cyclogenesis in the Arctic Ocean.

From the early 1980s to 2010s, the area of open water increased from around 10% to 40% in the Laptev, East Siberian, Chukchi and Beaufort Seas, which, in terms of the number of analyzed grid points of the ERA-Interim reanalysis, is an increase from around 500 to 2000 points. This four-fold increase in *N* results in about a 10% increase according to equation (2), assuming a Rayleigh distribution for simplicity. Subsampling the data by a factor of 10 to ensure independence from the neighbouring data increases this value to around 16%. Therefore, the larger the ice-free water area, the greater the probability of encountering a large wave. Physically, a large wave height must be associated with a strong wind speed. Indeed, the analysis reveals a clear trend in the increase of the expected value of area-maximum wind speed, which is highly correlated with the wave height.

Therefore, the emergence of ice-free water in the Arctic Ocean increases the chances of encountering a storm that would otherwise have occurred over the ice-covered sea. The changing ice-free water distance affects the wave field as well, but a complicated fetch geometry and an equivalent fetch need to be taken into consideration. With regard to the continuation of this trend, the effect of an increased encounter frequency stops as soon as the ice is completely lost from the sea during summer, with the remaining reason for the further change of the maximum wave height being the change in the storm conditions. However, in the past 40 years, storm activity does not have a clear positive trend. Moreover, whether the sea ice retreat continues until the ice is completely lost is not certain. Further studies on the air, sea, ice and wave interaction in the Arctic Ocean is warranted.

## Methods

### Expected value of the maximum significant wave height

Let *P*(*H*_*s*_) be the cumulative distribution function of the significant wave height *H*_*s*_,

8 $$P({H}_{s})={\int }_{0}^{{H}_{s}}p({H}_{s})d{H}_{s},$$

where *p*(*H*_*s*_) is the p.d.f. of the significant wave height *H*_*s*_. For *N* observational or grid points of a numerical wave model in ice-free waters, the largest significant wave height among these is

9 $$1-P({H}_{s}^{max})=\frac{1}{N}.$$

Assuming further that the p.d.f. can be approximated by a Weibull distribution $P({H}_{s})=\exp \{-{(\frac{{H}_{s}}{{c}_{{H}_{s}}})}^{k}\}$, the maximum significant wave height reads

10 $${H}_{s}^{max}={c}_{{H}_{s}}{(\mathrm{ln}N)}^{\frac{1}{k}},$$

which is valid for a large value of *N*^9^. This simple relationship indicates that the largest *H*_*s*_ in ice-free waters is related to the distribution of *H*_*s*_, the scale parameter ${c}_{{H}_{s}}$, and the extent of the ice-free waters as represented by *N*.

For a given number of *N*, the observed ${H}_{s}^{max}$ is itself a stochastic variable. Therefore, the most likely values of ${H}_{s}^{max}$ can be derived as follows. First, consider the probability that the significant wave height exceeds *H*_*s*_ at one observational point, or at one grid point, 1 − *P*(*H*_*s*_)^*N*^. Following Longuet-Higgins^27^, the probability that the maximum value of the significant wave height lies within *H*_*s*_ and *H*_*s*_ + *dH*_*s*_ is *dP*(*H*_*s*_)^*N*^ = *NP*(*H*_*s*_)^*N*−1^*p*(*H*_*s*_)*dH*_*s*_, and thereby, the p.d.f. of the ${H}_{s}^{max}$ is given as

11 $$f({H}_{s}^{max})=N\,P{({H}_{s})}^{N-1}p({H}_{s})\,d{H}_{s}.$$

If the cumulative distribution function *P* or the p.d.f *p* of *H*_*s*_ is known, the median, mode and mean of ${H}_{s}^{max}$ can be readily derived from *f*. In particular, the expectation or the mean of the ${H}_{s}^{max}$ can be expressed as

12 $$E[{H}_{s}^{max}]={\int }_{0}^{\infty }[1-P{({H}_{s})}^{N}]d{H}_{s}.$$

### Waves In Ice (WII) buoy system

The WII buoy system developed by P.A.S. Consultants is based on a system originally designed to measure the wave-induced motion of ice floes in the Antarctic marginal ice zone^28^. The nine degrees of freedom, three-axis gyroscope, accelerometer, and compass sensor (IMU MPU9250) is placed in a drifting buoy, and the data is processed on board by an Intel Edison processor. The motion is measured at 1-, 3- and 6-h sampling intervals for a 40-min data capture length starting at 5 min past the hour. When the battery voltage level lowers, the interval can be switched to a sleeping mode.

The accelerometer data is Fourier transformed, high-pass filtered and integrated to obtain various wave parameters. The details of the analysis method and the derived parameters are described in Kohout *et al*.^28^ The statistical quantities and power spectral density (p.s.d.), as well as the metadata, are transferred by Iridium satellite link to a server maintained by P. A. S. Consultants and is made accessible online. The raw data is also stored on a memory card.

The commercially available Sealite buoy was modified to include an internal electronics tube for the placement of three solar panels on its side, and to attach a 15-kg steel weight at the bottom of the keel (Fig. S2) for a total weight of about 35 kg. The battery lasts for 3 weeks without charging. Integrated air, sea and internal temperature probes are also included. Further details are given in Waseda *et al*.^14^.

### Accelerometer data processing

The low-frequency noise of an accelerometer record is commonly removed with an ideal high-pass filter. Because the WII system was originally developed for measurements in the Antarctic Ocean which is unbounded, and hence accounts for waves of much longer wave period than that found in the Arctic Ocean, the on-board processing applies a high-pass filter at a 33-s cutoff period. The mean of the p.s.d. of buoy 1 for the entire period is shown in Fig. S3. As the signal-to-noise ratio is large at a much higher frequency than the cutoff frequency of 33 s, the significant wave height based on a spectral moment is overestimated. As a remedy, different cutoff frequencies are used when comparing with the ERA-Interim data. With a 15-s cutoff frequency, the correlation coefficient for the significant wave height is 0.91 for buoy 1 and 0.9 for buoy 2, while the correlation coefficient of the mean wave period is 0.15 for buoy 1 and 0.2 for buoy 2. By changing the cutoff frequencies to 10 s, the correlation coefficients im prove to 0.68 for buoy 1 and 0.63 for buoy 2. We have then imposed an adaptive cutoff frequency filter such that the local minima of the smoothed spectra at each time step are used as the cutoff frequency. The correlation coefficients of the mean wave period further improve to 0.78 for buoy 1 and 0.76 for buoy 2 (Fig. S4). Note that different moment periods were compared and the energy period defined as *T*_0,−1_ = (*m*_0_/*m*_−1_)^−1^ agreed best with the reanalysis. Further details are given in Waseda *et al*.^14^.

### Data Availability

The observed wave data analysed during the current study are available in the Arctic Data archive System (ADS) repository, https://ads.nipr.ac.jp/dataset/A20180306-001.

## Electronic supplementary material

Supplementary Materials
