# Supplementary material for: Correlated Increase of High Ocean Waves and Winds in the Ice-Free Waters of the Arctic Ocean
Source: Sci Rep. 2018 Mar 14;8:4489. doi: 10.1038/s41598-018-22500-9 (PMC5852140; doi:10.1038/s41598-018-22500-9)
Supplement: Supplementary file 1 — Supplementary Materials [file 41598_2018_22500_MOESM1_ESM.pdf]

Correlated Increase of High Ocean Waves and Winds in the Ice-Free Waters of the Arctic Ocean - Supplementary Materials

Takuji Waseda[1*], Adrean Webb[1,5], Kazutoshi Sato[2,6], Jun Inoue[2], Alison Kohout[3], Bill Penrose[4], and Scott Penrose[4]

[1]The University of Tokyo, Graduate School of Frontier Sciences, Kashiwa, 277-8563, Japan
[2]National Institute of Polar Research, Arctic Environment Research Center, Tachikawa, 190-8518, Japan
[3]National Institute of Water and Atmospheric Research, Hydrodynamics, Christchurch 8011, New Zealand
[4]P.A.S. Consultants Pty Ltd., Panton Hill, 3759, Australia
[5]Kyoto University, Disaster Prevention Research Institute, Kyoto, 611-0011 Japan
[6]Kitami Institute of Technology, Kitami, 090-8507, Japan
[*]waseda@k.u-tokyo.ac.jp

ABSTRACT

The supplementary materials include figures and tables referenced from the Results section and the Methods section.

Results

Validation of the reanalysis wave field

Large wave events were observed, for example, on 19 September, when buoys 1 and 2 recorded a H_s of 4.86 m and 4.63 m, respectively, which is comparable to the highest wave directly observed in this region by Thomson et al.[1] During this time, an Arctic cyclone developed off Point Barrow (Fig.S1)[2] to the north of the buoy locations. As the location of the large wave height was recorded south of the cyclone centre, which is relatively widespread, the largest significant wave height in this region may even have been larger than the buoy observation. The H_s of the ERA-Interim data and the buoys agree quite well during this storm.

The buoy observations were used to validate the ERA-Interim reanalysis. The error metrics of ERA-Interim wave parameters, the significant wave height, and the mean periods are summarized in table S1. The normalized bias (NBIAS), root mean square error (RMSE), correlation coefficient (C.C.) and the normalized standard deviation (NSTD) were estimated (see e.g. Stopa et al. (2016) for the definitions).[3]

Table S1. Error metrics of ERA-Interim wave field (H_{m0} and T_m) against buoy observations

	NBIAS	RMSE	SI	C.C.	NSTD
Hm0: Buoy1	0.61 %	0.088 m	29.5 %	0.94	-20.2 %
Hm0: Buoy2	-2.89 %	0.068 m	27.9 %	0.89	-22.9 %
T0m1:Buoy1	-8.54 %	0.64 s	10.1 %	0.81	6.2 %
T0m1:Buoy2	-11.27 %	0.75 s	10.0 %	0.78	12.3 %

The trend of the expected largest significant wave height in the ice-free waters

The robustness of the trend was tested using the homogenization tool developed by Wang and Feng (2013)[4,5]. The analysis was conducted without the reference series with a confidence level of 0.95. None of the known transitions from the assimilation-free period to the assimilated period and back to the assimilation-free period in 1991 and 2011 respectively, were detected by the initial investigation. Then, these years were given as a known transition, which yielded the mean shifted trend estimates shown in Fig. S2. Except for August where the adjusted trend decreased from 0.011 m/year to 0.003 m/year, the trend increased with the adjustments: September trend increased from 0.011 m/year to 0.012 m/year, October trend increased from 0.02 m/year to 0.024 m/year. Therefore, September and October trend estimate is robust whereas the August trend requires some further

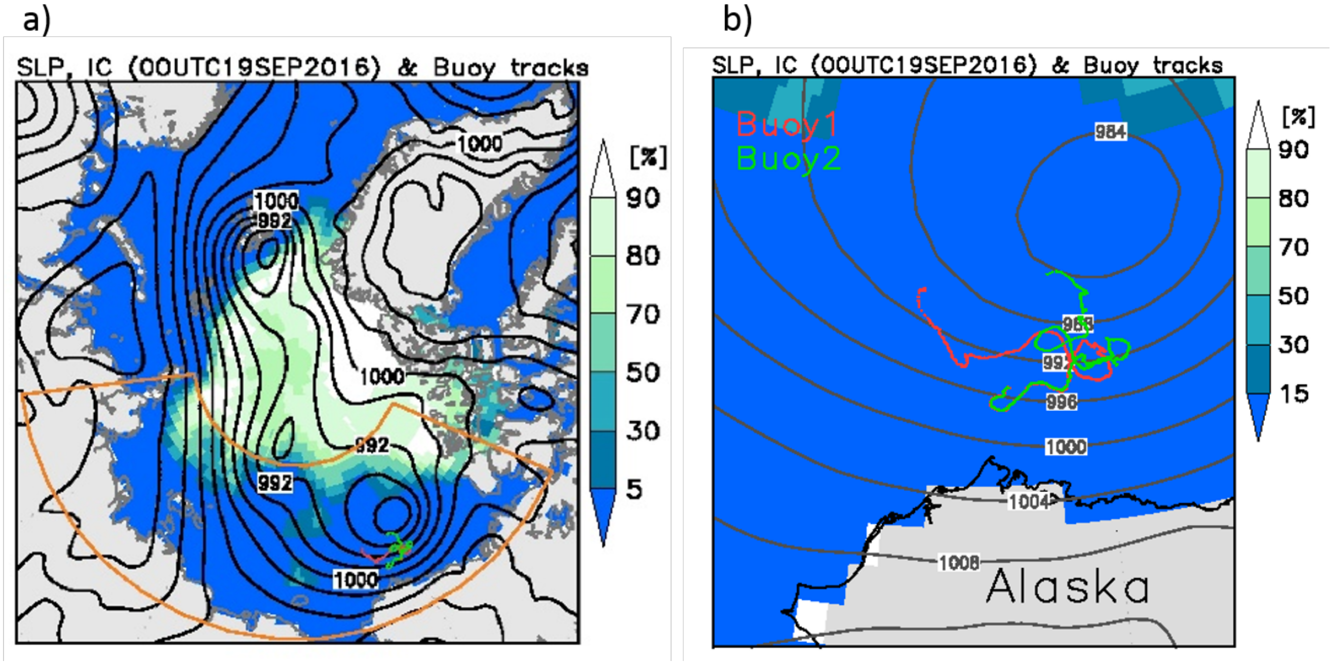

Figure S1. (a) Buoy trajectories, sea-ice concentration (colour shading) and sea-level pressure (SLP) (contours) on 19 September 2016. The region bounded by the orange line indicates the area analyzed here. (b) An enlarged image of the buoy trajectories with the sea-ice concentration (colour shading) and SLP (contours) on 19 September 2016. The SLP and sea-ice concentration are from ERA-Interim reanalysis data. The Grid Analysis and Display System (GrADS) version 2.0.2 (http://cola.gmu.edu/grads/) was used to create the maps in this figure.

investigation. It should also be noted that no obvious step-like transition related to change of the ice concentration assimilation methods was seen. There were no step-like transition detected in the wind speed as well.

Methods

Waves In Ice (WII) buoy system

The WII buoy system developed by P.A.S. Consultants is shown in Fig. S3. The commercially available Sealite buoy was modified to include an internal electronics tube for the placement of three solar panels on its side, and to attach a 15-kg steel weight at the bottom of the keel for a total weight of about 35 kg. The battery lasts for 3 weeks without charging. Integrated air, sea and internal temperature probes are also included. Further details are given in Waseda et al.[2].

Accelerometer data processing

The low-frequency noise of an accelerometer record of the WII system applies an adaptive cutoff period detecting the minimum of the surface elevation spectrum at each observation. The mean of the p.s.d. of buoy 1 for the entire period is shown in Fig. S4. When the original cutoff frequency of 33 s is used, spectral moments becomes erroneous. Different cutoff frequencies were tested comparing with the ERA-Interim data. With a 15-s cutoff frequency, the correlation coefficient for the significant wave height is 0.91 for buoy 1 and 0.9 for buoy 2, while the correlation coefficient of the mean wave period is 0.15 for buoy 1 and 0.2 for buoy 2. By changing the cutoff frequencies to 10 s, the correlation coefficients improve to 0.68 for buoy 1 and 0.63 for buoy 2. The correlation coefficients of the mean wave period further improve to 0.78 for buoy 1 and 0.76 for buoy 2 when an adaptive cutoff frequency is used (Fig. S5). Different moment periods were compared, $T_{01} = m_0/m1$, $T_{02} = (m_0/m_2)^{1/2}$, $T_{0,-1} = (m_0/m_{-1})^{-1}$, $T_{24} = (m_2/m_4)^{1/2}$, and the energy period defined as $T_{0,-1}$ agreed best with the reanalysis. Here, m_n represent spectral moments. Further details are given in Waseda et al.[2].

Data availability

The observed wave data analysed during the current study are available in the Arctic Data archive System (ADS) repository, https://ads.nipr.ac.jp/dataset/A20180306-001

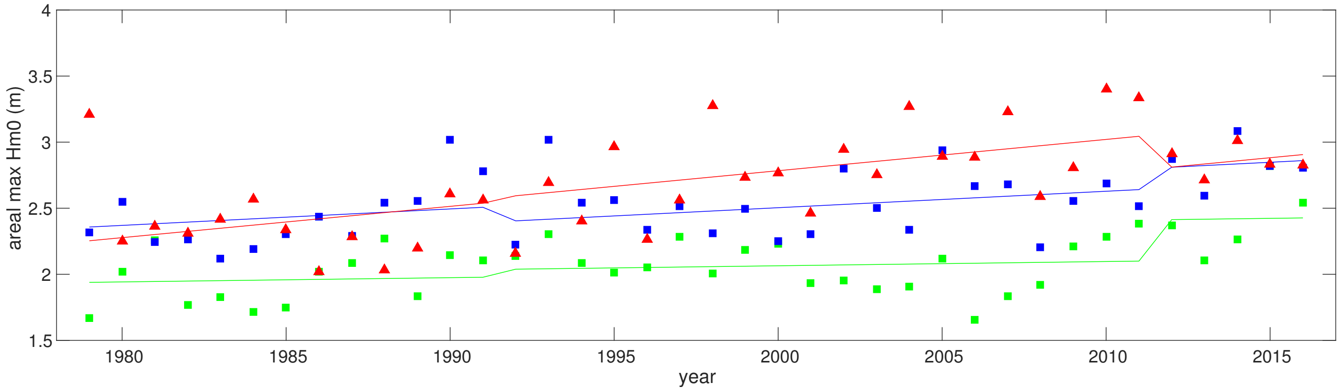

Figure S2. The annual $E\left[H_s^{max}\right]$ are plotted for August (●), September (■) and October (▲) from 1979 to 2016. The linear trends with mean adjustments in 1991 and 2011 are indicated by green (August), blue (September) and red lines (October).

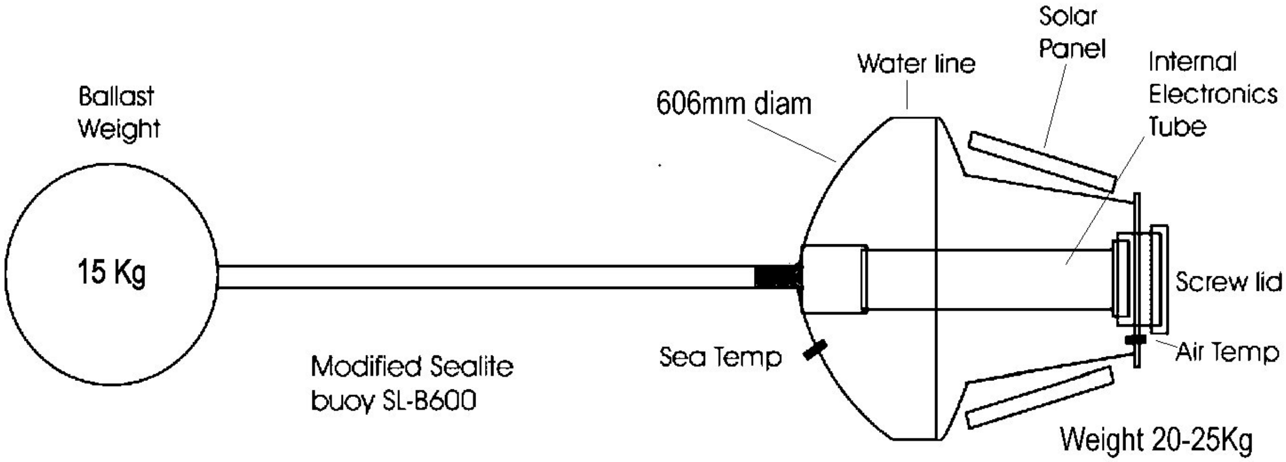

Figure S3. Schematic diagram and image of the WII buoy, shown side ways. Three external solar panels are used with 7.2 V, 60 Amp hour battery. Nine degrees of freedon IMU unit and Intel Edison processor are used. Further technical questions regarding the WII buoy should be addressed to bill.penrose@pas.com.au

References

1. Thomson, J. Onr sea state dri cruise report r/v sikuliaq, fall 2015. `http://www.apl.washington.edu/project/projects/arctic_sea_state/pdfs/cruise_report.pdf` (2015). Accessed on 2018-1-6.
2. Waseda, T. *et al.* Arctic wave observation by drifting type wave buoys in 2016. In *The 27th International Ocean and Polar Engineering Conference* (International Society of Offshore and Polar Engineers, 2017).
3. Stopa, J. E. Wave climate in the arctic 1992-2014: seasonality and trends. *The Cryosphere* **10**, 1605 (2016).
4. Aarnes, O. J., Abdalla, S., Bidlot, J.-R. & Breivik, Ø. Marine wind and wave height trends at different era-interim forecast ranges. *J. Clim.* **28**, 819–837 (2015).
5. Wang, X. L. & Feng, Y. Rhtestsv3 user manual. *Clim. Res. Div. Atmospheric Sci. Technol. Dir. Sci. Technol. Branch, Environ. Can.* **24** (2010).

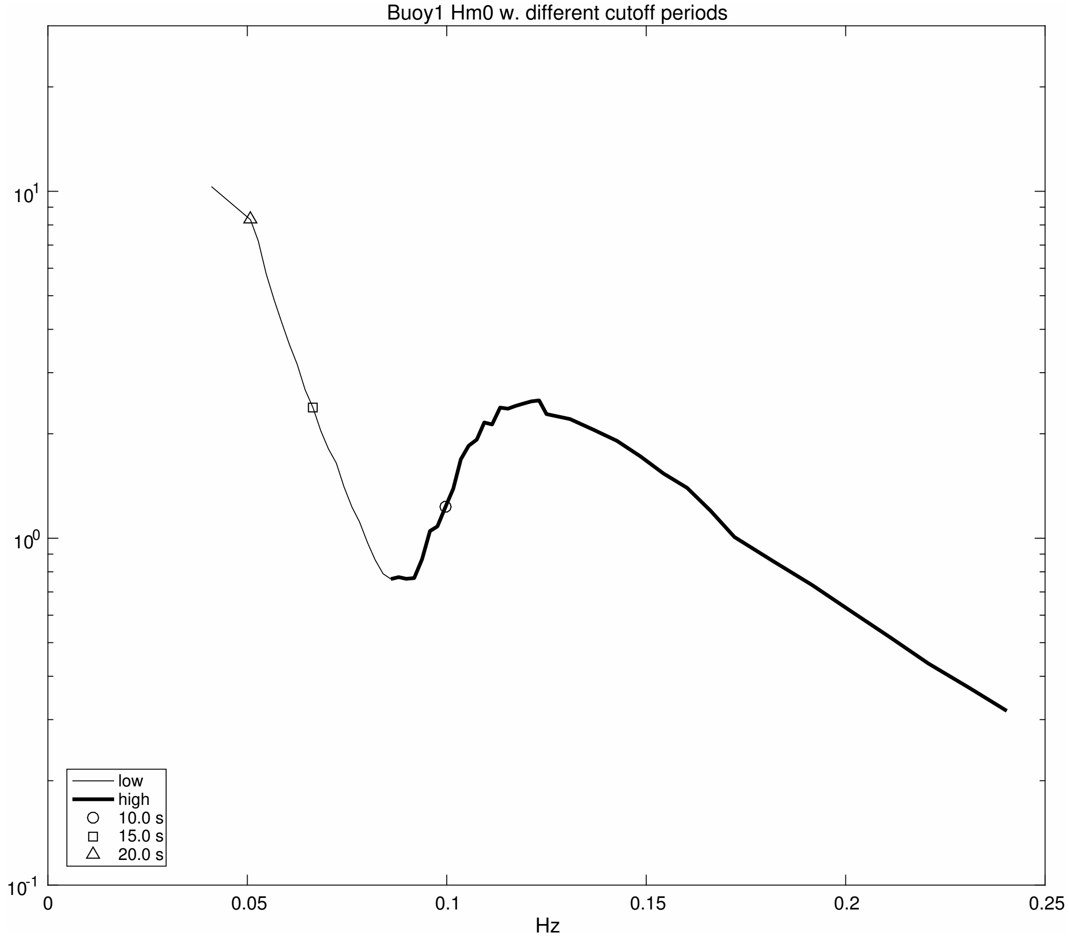

Figure S4. The mean power spectral density from buoy 1.

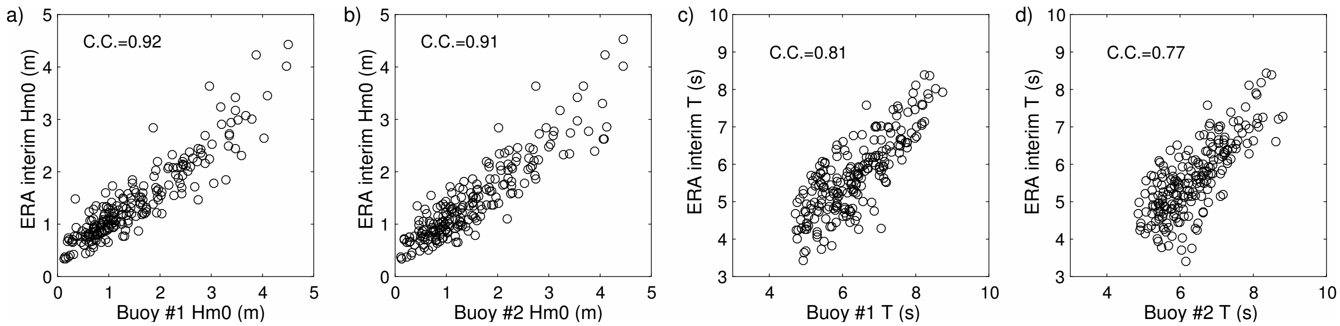

Figure S5. Top row: scatter plots of significant wave heights of buoy observations and ERA-interim reanalysis data. Bottom row: scatter plots of mean wave periods. An adaptive cutoff frequency based on each power spectral density is used.
